# Case report: Organ donation after euthanasia for psychiatric suffering: some of the practical and ethical lessons Martijn taught us

**DOI:** 10.3389/fpsyt.2024.1234741

**Published:** 2024-03-05

**Authors:** Nathalie van Dijk, Wim de Jongh, Paulan Stärcke, David Shaw, Jan Bollen, Walther van Mook

**Affiliations:** ^1^ Department of Intensive Care Medicine, Maastricht University Medical Center+, Maastricht, Netherlands; ^2^ Department of Organ Donation Coordination, Maastricht University Medical Center+, Maastricht, Netherlands; ^3^ Expertisecenter Euthanasia, The Hague, Netherlands; ^4^ Care and Public Health Research Institute, Maastricht University, Maastricht, Netherlands; ^5^ Institute for Biomedical Ethics, University of Basel, Basel, Switzerland; ^6^ Department of Anesthesiology, Pain and Palliative Medicine, Radboud University Medical Center, Nijmegen, Netherlands; ^7^ Academy for Postgraduate Training, Maastricht University Medical Center+, Maastricht, Netherlands; ^8^ School of Health Professions Education, Maastricht University, Maastricht, Netherlands

**Keywords:** euthanasia, organ donation, psychiatric suffering, case report, assisted suicide and euthanasia

## Abstract

Euthanasia in psychiatric patients presents unique challenges, especially when combined with organ donation. In this article, the hurdles psychiatric patients might encounter after expressing their wish for organ donation after euthanasia, are discussed and illustrated by the case of Martijn, a 45-year-old psychiatric patient who altruistically donated his organs after euthanasia. Hospital and physician-related factors, including caution in determination of mental capacity, consideration of conflicting interests, and healthcare staff stress are discussed as impediments to organ donation after euthanasia (ODE) in psychiatric patients. The primary objective of this article is to raise awareness among psychiatrists regarding the fact that although the combination of euthanasia and organ donation is an uncommonly performed procedure, it is frequently requested by psychiatric patients. In conclusion, the article advocates for a nuanced approach, respecting patients’ altruistic wishes while at the same time addressing challenges associated with ODE in psychiatric suffering. Where possible, and within the current medical, ethical and legal boundaries, the importance of facilitating organ donation without unnecessarily prolonging the suffering of competent psychiatric patients seeking euthanasia is emphasized. The topic calls, for example, for further qualitative research to understand the stakeholders’ perspectives to determine the perceived possibilities on the one hand and boundaries on the other.

## Introduction

### Euthanasia in psychiatric patients

Euthanasia is legalized in Belgium, the Netherlands, Luxembourg, Colombia, Canada and parts of Australia (1–4). In 2021, euthanasia was permitted in Spain and New Zealand. In the Netherlands euthanasia was legalized by the Termination of Life on Request and Assisted Suicide Act in 2002. Since then, euthanasia has been allowed following an explicit, voluntary and well-considered request, when the patient is suffering with no prospect of improvement and with no reasonable alternative. In addition, an independent physician must also give an opinion on compliance with all due care criteria.

Between 2002 and 2022, 12,1867 patients underwent euthanasia in the Netherlands, most commonly because of end-stage cancer (5).

In 1994, euthanasia in a patient suffering from psychiatric illness was facilitated for the first time. The Dutch Supreme Court ruled that unbearable and irreversible psychiatric suffering justified euthanasia, but mandated consultation of a second independent psychiatrist (6). Specific due diligence criteria are currently included in the Euthanasia Code 2022 of the Regional Euthanasia Review Committees (RTEs) as well as the Dutch Society of Psychiatrists’ guideline (7, 8). In case of psychiatric suffering, an opinion by a psychiatrist competent to assess the patient’s specific pathology is mandatory, to optimally assess the patient’s decision-making competence regarding the request for euthanasia, to confirm the lack of prospect of improvement and the absence of reasonable alternatives. Furthermore, even if the attending physician is a psychiatrist, it is still essential to involve a second independent psychiatrist as additional assessor (7).

A mental disorder is defined in DSM-5-TR as a syndrome characterized by clinically significant disturbance in an individual’s cognition, emotion regulation, or behavior that reflects a dysfunction in the psychological, biological, or developmental processes underlying mental functioning. Mental disorders are usually associated with significant distress or disability in social, occupational, or other important activities. An expectable or culturally approved response to a common stressor or loss, such as the death of a loved one, is not a mental disorder. Socially deviant behavior (e.g. political, religious, or sexual) and primary conflicts between an individual and society are formally not classified as mental disorders unless the deviance or conflict results from a dysfunction in that individual, as described above (9). Euthanasia in psychiatric suffering is only permitted when the patient has maximally pursued all reasonably possible treatment options, through which one can also evaluate the patient’s perseverance regarding the request. In the Netherlands, in 2022, euthanasia was performed in 8720 patients, with 115 patients (1.3%) primarily suffering from a psychiatric illness (10). Around 10% of euthanasia requests in psychiatric patients are reportedly granted (11). Around 80% of requests for euthanasia in psychiatry are assessed by the Expertisecenter Euthanasia (EE) (12). The number of requests for euthanasia from patients suffering from psychiatric disorders at the EE was 781 in 2022, 18.8% of the total number of requests. For 90 patients with psychiatric suffering, these requests were ultimately granted (11.5%) (13). Euthanasia in psychiatric patients appears to occur infrequently due to the complexity of assessing the due care criteria. The waiting time for the EE currently exceeds 2.5 years from registration to the initial assessment interview for cases that require examination by a team which includes a psychiatrist. Thereafter, the investigational process itself takes a minimum of three months, but usually longer, contingent upon the complexity of the case (personal communication EE). Each euthanasia case is reviewed *post hoc* by a Regional Euthanasia Review Committee (RTE) consisting of a lawyer, an ethicist and a physician within six weeks after the procedure.

### Organ donation after euthanasia

Organ donation after euthanasia is currently performed in Belgium, the Netherlands, Canada Spain and parts of Australia. Neither the Dutch law on organ donation nor the Dutch Termination of Life on Request and Assisted Suicide Act precludes organ donation after euthanasia. The subject of organ donation should preferably be raised by the patient after the euthanasia request is approved to avoid the euthanasia request arising from the wish for donation. This sequence, as documented in the National Dutch Organ Donation after Euthanasia guideline, aims to ensure a clear separation of the euthanasia assessment and subsequent organ donation request (14). Nevertheless, this current guideline acknowledges that patient care should always be patient-tailored, individualized care. In the national Dutch opt-out system introduced in July 2020 (15), all patient preferences regarding donation are documented. In the absence of a patient’s registered active refusal to donate, the patient’s (presumed) consent may scaffold further dialogue facilitating shared decision-making while respecting the patient’s decisive autonomy regarding organ donation after euthanasia.

The first ODE procedure in the Netherlands was performed in 2012 (14). Organ donation after euthanasia is a donation after circulatory death (DCD) procedure (16), which can only be performed in a hospital. DCD is medically possible in the absence of medical contraindications, after fulfilling all criteria in the Dutch Organ Donation Act, and following the death determination guideline by the Dutch Health Council (17). Malignancy is the most common general contraindication for donation. Of all patients who undergo euthanasia 10% are estimated to be potentially medically eligible to donate (18). Most commonly eligible patients suffer from neurodegenerative diseases, e.g. amyotrophic lateral sclerosis (ALS) or multiple sclerosis (18). Until April 2023, more than one hundred patients donated their organs following euthanasia in the Netherlands, including their lungs, kidneys, pancreas, liver and since March 2021, their heart (19, 20). More detailed information about the practical aspects and governance of ODE can be found elsewhere (14).

### Organ donation after euthanasia in psychiatric patients

Currently, ODE in psychiatric patients is only briefly touched upon in the Dutch ODE guideline (21). This may be at least in part attributable to the fact that experiences with organ donation after euthanasia in patients suffering from a psychiatric disease have been described and systematically explored to only a limited extent (22). Studies that provide insights into the reasons why psychiatric patients choose euthanasia and how this relates to the option of suicide are limited. One reason given for choosing euthanasia over suicide is the opportunity to donate organs (23). This article specifically focuses on the unique considerations and challenges associated with psychiatric patients making a request for euthanasia and organ donation. We herein describe the unique illustrative case of Martijn, a psychiatric patient who donated his organs after euthanasia. After describing the case of Martijn, this article will discuss the difficulties Martijn faced during his journey to euthanasia and subsequent donating his organs and also the difficulties the healthcare personnel faced regarding ODE in the case of Martijn.

## Case description

Martijn, who reached the age of 45, suffered from several psychiatric disorders since he was a teenager, including borderline personality disorder, attention deficit hyperactivity disorder (ADHD) and substance abuse. His family history revealed a grandfather with bipolar disorder.

His father left the family when Martijn was six years old, his mother remarried and when he was twelve years old, he adopted the name of his stepfather. In his teens, he blamed himself for the divorce. During this period, he had more and more trouble containing his aggression, had few friends in high school, and was bullied for his protruding ear. At the age of 19, he underwent psychotherapy to temper outbursts. After he completed his military service at 20 years of age he started drinking rather heavily. He had a few unsuccessful relationships, after one of which he attempted to commit suicide and was admitted for crisis intervention. After his second relationship, he stopped drinking alcohol, but continued to use marihuana on a daily basis.

For three decades, he was unsuccessfully treated with different treatment regimens, both medically as well as psychotherapeutic interventions (e.g. individual psychoeducation, addiction treatments, aggression-regulation training, social psychiatric support and psychotherapeutic intervention for personality issues). He suffered from his underlying psychiatric disorders and failure of treatments, but also from associated life events, such as failure to be successful regarding employment, and personal relationships. He was chronically depressed and was desperately longing to end of his suffering. Despite his strong and persisting wish to die, this intelligent man did not want his relatives to suffer the consequences of (non-assisted) suicide. He voiced his wish to die peacefully, and in addition, his altruistic wish to be able to help others – by donating his organs.

His treating physician objected to performing euthanasia, even though he fulfilled all due diligence requirements. He was referred to the Expertisecenter Euthanasia (EE), where more than three conversations took place over a period of a year and a half, after which the EE psychiatrist consented to perform euthanasia. The psychiatrist concluded that he suffered from a low-level integrated personality disorder, for which further therapy was unlikely to be successful, all the more so since he was tired of therapy and had no motivation anymore. Several Dutch hospitals, including the university hospital near his home, refused to facilitate ODE. Consultations between the working groups of national coordinating transplant coordinators and intensive care physicians ultimately resulted in approval for the combined procedure by a university medical center 220 kilometers from his home address, which in the Netherlands, was considered a long distance. He was extremely relieved when he discovered organ donation after euthanasia was possible, which was strongly supported by his relatives and friends. Martijn subsequently died on day 16,514 of his life, with a smile on his face, with his final words being: “*It is okay*”.

## What is known about ODE in patients suffering from a psychiatric disorder?

The first case of ODE in a patient suffering from a psychiatric disorder dates from 2013. Since 2016, the number of cases has increased annually. In 2020, the number of patients that underwent ODE for psychiatric disease transcended that of somatic disorders (24). Over recent years, interest in patients’ journeys such as Martijn’s has grown significantly (25). Its impact on the number of requests for ODE remains unclear. Until January 1^st^ 2022, 24 patients suffering from an underlying psychiatric disorder chose to donate their organs following euthanasia (from a total of 85, 27%) (24). On January 1^st^ 2023, 29 out of 98 ODE patients had a psychiatric disease (29.6%). Patients with ODE based on an underlying psychiatric disease were 5 years younger than the average population of patients who underwent ODE (48.8 years versus 53.8 years) (24). Patients who underwent ODE also appeared to be younger than the general euthanasia population, although an average age for the latter is not available (26). However, the vast majority of all euthanasia patients in 2020 until 2022 (87.6%, 89% resp. 89%) were aged over 60 years of age (26–28). The same study reported that 1.1% of somatic patients donated their organs after euthanasia. In comparison, the percentage of patients with an underlying psychiatric disorder that underwent organ donation after euthanasia (24/634; 3.8%) was significantly higher (24).

## Practical hurdles regarding organ donation after euthanasia for psychiatric patients

Organ donation after euthanasia in psychiatric suffering is surrounded by several mainly ethical and practical challenges, related to euthanasia on the one hand, but also to the subsequent organ donation procedure on the other.

In the sections below, we consecutively discuss the different factors that contributed to the difficulties Martijn faced separately, although acknowledging that in practice, these factors are more intertwined: hospital and physician-related factors, caution exercised regarding the determination of competence, consideration of conflicting and intertwined interests and discomfort and stress for healthcare staff.

### Hospital and physician’s factors

In 2022, the assistance of physicians from the euthanasia expertise center was invoked more frequently (78%) when the euthanasia requests involved psychiatric patients in comparison to euthanasia requests by patients suffering from all other clinical conditions in which the expertise center’s support was sought (10). Many psychiatrists find euthanasia in psychiatric patients challenging due to moral, epistemological, practical, and contextual problems it presents. In general, this has been shown to lead to a reserved attitude toward euthanasia in psychiatric patients (29, 30).

### Why was it so difficult for Martijn to find a hospital supporting his request?

Not all hospitals and/or physicians in the Netherlands are willing to honor a patient’s wish for organ donation after euthanasia. There is a striking geographical predominance of ODE in psychiatric illness in the East Netherlands (24). There is a continuum of hospitals, running from those that do not facilitate ODE at all regardless of underlying disease, hospitals that are willing to facilitate ODE but only in patients from their own region and/or those with whom a treatment relationship already existed, hospitals that facilitate ODE in somatic patients, yet *not* in patients suffering from psychiatric disorders, to hospitals that are willing to facilitate ODE in all categories of patients, regardless of their underlying disease (24). The latter willingness is in line with the January 2023 revised national ODE guideline in which a distinction between underlying causes of suffering is no longer made (21).

Data which provide insights into the reasons for this observation are lacking and can only be speculated about. Hospitals may be afraid of being prosecuted (wrongly) and of possible negative publicity. Some hospitals, therefore, assess the detailed content underlying each euthanasia request again in order to minimize any risk of culpability, even though due diligence criteria have already been examined by the euthanizing physician as well as the second and third independent physicians (in compliance with the previously mentioned Euthanasia Code 2022 of the Regional Euthanasia Review Committees (RTEs) as well as the Dutch Society of Psychiatrists’ guideline). This leads to further delay and prolongation of the patient’s suffering. However, as the current Dutch guideline on ODE clearly states, the euthanasia procedure needs to be strictly separated from the organ donation procedure, as the transplantation procedure needs to be strictly separated from the donation procedure (21).

The staff involved in the request for organ donation by a psychiatric patient whose request for euthanasia has already been granted, should, in our opinion, not put themselves in the shoes of those formally responsible for the euthanasia assessment procedure and its outcome. This does not preclude greater caution regarding competency to be warranted and attributed to patients with mental conditions.

To date, *post hoc* analysis confirmed procedural correctness in all cases of euthanasia for psychiatric suffering since 2002, despite the fact that per-procedural differences in physicians’ opinion regarding competence were identified in 0-12% of cases in two case series between 2011-2014 (31) and 2015-2017 (32). Prosecution has so far not occurred.

A recent cross-sectional study on the rates of euthanasia (not followed by organ donation) also revealed considerable geographical variation across the Netherlands (33), however with predominance of euthanasia performed in the Western part. Factors associated with this Western predominance were, for example, age, church attendance, political orientation, income, and self-experienced health. After adjustment for these characteristics, a considerable amount of geographical variation remained, which warrants further exploration, comparable to ODE in psychiatric illness.

### Caution exercised regarding determination of competence

Secondly, a euthanasia request needs to be well-considered, and thus the patient needs to be mentally competent, which has to be evaluated multiple times by the treating physician and independent physician. In psychiatric suffering particular caution must be exercised when assessing the patient’s decisional competence with regard to their request for euthanasia and their request for organ donation. The emphasis on the strict separation of euthanasia and organ donation procedures is crucial and consequently it needs to be thoroughly investigated whether the desire for euthanasia has in no way stemmed from a wish to donate organs. The physician must rule out that the patient’s power of judgment is impaired by their psychiatric disorder(s). If patients are not mentally competent with regard to their requests for euthanasia and/or organ donation, these requests cannot be regarded as voluntary and well considered. The physician must take particular note of whether the patients are able to grasp relevant information, understand their disease and are unequivocal in their deliberations. In general, greater caution regarding competency is warranted and attributed in patients with mental conditions. However, the fact that a patient suffers from a psychiatric disorder should not be used simply as an excuse to deny them access to euthanasia, nor to permit them access to euthanasia, but deny them access to ODE (34).

### Consideration of conflicting and intertwined interests

Thirdly, guardians and caregivers want to avoid situations where patients request euthanasia because they are able to donate their organs, an argument more generally voiced by opponents of the combined procedure. As mentioned, the percentage of patients with an underlying psychiatric disorder who underwent organ donation after euthanasia (3.8%) compared to the percentage of patients who donated after euthanasia based on an underlying somatic disorder (1.1%) from 2012-2020 was remarkably high (24). A previous publication speculated that patients suffering from psychiatric illness may be more altruistic, or have become more altruistic, due to reflections about their lives, or private experiences with organ donation, and consequently want to finalize their lives with an altruistic gift to for them unknown others (24). Another possible explanation is that psychiatric patients, e.g. in comparison to ALS patients, are physically less hindered regarding information gathering, e.g. on the internet, on the possibilities of organ donation after euthanasia, or to undergo the for donation necessary preparatory examinations. Another contributory factor is perhaps the longer time frame between the euthanasia requests and its performance in psychiatric patients, compared to somatic patients. Also, at least theoretically, the euthanizing physicians involved in euthanasia in psychiatric patients could have more frequently raised the topic of organ donation, in comparison to somatic patients. However, the Dutch ODE guideline does not favor proactively raising the option of donation after euthanasia by healthcare staff in general. Finally, psychiatric patients might assume, more frequently than somatic patients, that their organs are medically suitable for donation, and thus raise the topic of ODE more frequently. This assumption is not quite justified however, considering the high prevalence of adverse health behaviors, including tobacco smoking, other substance use, physical inactivity, and poor diet in people with mental disorders, compared to the general population (35). The limited data on ODE in psychiatric patients in general, and the lack of outcome data after transplantation precludes any conclusions regarding the explanations postulated.

### Discomfort and stress for the healthcare staff

Another important aspect to consider is the impact of organ donation after euthanasia in psychiatric patients on the well-being of health care professionals involved. In a patient who requests euthanasia because of physical suffering, the underlying somatic illness and associated suffering are often readily observable, and death is imminent in the foreseeable future. The associated moral distress with the same procedure in a psychiatric patient, in which the illness is often not directly observable, and which, on average, is 5 years younger, may be substantially higher. This is currently being investigated through an interview study involving healthcare professionals who have participated in an ODE procedure with a psychiatric patient. Furthermore, it can be envisioned that symptoms of some psychiatric disorders could potentially worsen due to the stressful pre-admission preparatory visits and admission and may cause additional discomfort for the healthcare professionals and the patient’s relatives. This may warrant precautionary conversations between the patients and the healthcare professionals, to weigh the interests of the patient against those of other patients, as well as the interests of their relatives, the team, hospital and society. Furthermore, performing several such procedures within a short time frame, can exhaust the team’s capacity to optimally perform and support, and consequently result in refusal of a subsequent request, despite a team’s willingness and positive stance towards ODE in this group of patients.

### Other considerations

From the perspectives of the psychiatrists involved, we have learned that these procedures necessitate careful consultations between the general practitioner, euthanizing physician, organ donation coordinator and intensive care staff on a local and regional level. When a specific patient’s request for ODE meets resistance in one hospital because, transiently or permanently, the patient’s and the hospital staff’s interests do not align, concerted efforts of regional organ donation coordinators and regional coordinating intensivists for donation affairs may be warranted in order to attempt to respect the patients’ altruistic last wish to donate their organs. So far, every patient and procedure performed has its own unique context and timing because of which it is challenging to create generalized guidelines for ODE in psychiatric illness. The above-mentioned factors however do shed some preliminary light onto this so far hardly explored topic. Due to its focus and word count restraints, this article does not include a comprehensive discussion of ethical considerations associated with ODE procedures in psychiatric patients. A more in-depth ethical dialogue is however necessary, and future quantitative and qualitative research addressing the various (practical and ethical) perspectives of the different stakeholders, is essential to provide more insight into these aspects related to the care of the subset of psychiatric patients who requested ODE. Consequently, a survey study is planned among healthcare professionals involved in ODE, as well as a qualitative individual interview study involving healthcare professionals involved in ODE procedures for psychiatric patients, in which both the practical challenges and ethical considerations will be explored more in-depth. Last and foremost, the patients’ and relatives’/friends’ perspectives are paramount to be explored.

## Conclusions

Patients, such as Martijn, can suffer unbearably and hopelessly from psychiatric disorders, suffering that is different from, yet also comparable to patients with physical underlying disorders. Extra careful, strict criteria for the assessment of competence regarding the euthanasia requests in psychiatric patients are already in place. Any wish to donate organs after euthanasia, somatic and psychiatric patients alike, is an extremely altruistic act, and should be subject of careful and deliberate consideration. Any conflicts of interest and in perspective of each patient’s unique context should be critically considered. After completing the careful and often prolonged assessment process regarding euthanasia, the suffering of competent patients like Martijn should not be prolonged by refusals to deny them euthanasia, whether in isolation or in combination with organ donation. On the other hand, psychiatric patients (and somatic patients alike) should always feel free to decline euthanasia and/or organ donation at any time, for any reason, without experiencing any external pressure and/or discomfort.

Comparable to the EE, that serves as a safety net for physicians who cannot or do not wish to perform euthanasia, a safety net for (psychiatric) patients in whom the wish for euthanasia has been approved, but were unable to find a nearby hospital willing or able to participate in the combined procedure, should preferable be in place. Recently such safety net was established *ad hoc* for individual patients through collaboration of the regional coordinating donation intensivists of the Organ Donation Committee of the Dutch Association for Intensive Care to facilitate the ODE procedures in these patients. Such a concerted, sometimes thus national effort by all stakeholders may be warranted in order to enable respecting psychiatric patients’ altruistic last wishes to donate their organs after euthanasia.

## Data availability statement

The original contributions presented in the study are included in the article/supplementary material. Further inquiries can be directed to the corresponding author.

## Ethics statement

Ethical approval was not required for the study involving humans in accordance with the local legislation and institutional requirements. Written informed consent to participate in this study was not required from the participants or the participants’ legal guardians/next of kin in accordance with the national legislation and the institutional requirements. Written informed consent was obtained from the individual(s) for the publication of any potentially identifiable images or data included in this article.

## Author contributions

WM, JB and ND drafted the manuscript. ND, WJ, PS, WM treated Martijn and worked with the family. DS added ethical knowledge and expertise to the article. WJ contacted the parents for approval and coordinated the approval of the final draft. All authors contributed to the article and approved the submitted version.
